# Influence of maternal body mass index on pregnancy complications and outcomes: a systematic review and meta-analysis

**DOI:** 10.3389/fendo.2024.1280692

**Published:** 2024-06-04

**Authors:** Yi Zhang, Mei Lu, Ying Yi, Luming Xia, Renjun Zhang, Chao Li, Ping Liu

**Affiliations:** ^1^ College of Public Health, Zunyi Medical University, Zunyi, China; ^2^ Key Laboratory of Maternal & Child Health and Exposure Science of Guizhou Higher Education Institutes, Zunyi, China; ^3^ Qingdao Municipal Center for Disease Control and Prevention, Qingdao Institute of Prevention Medicine, Qingdao, China; ^4^ Center for Animal Disease Control and Prevention of Shanghai, Shanghai, China; ^5^ Center for Animal Disease Control and Prevention of Guizhou Province, Guiyang, China; ^6^ China Animal Health and Epidemiology Center, Qingdao, China

**Keywords:** maternal, body mass index, obesity, pregnancy outcomes, complications

## Abstract

**Background:**

The prevalence of obesity among women of reproductive age is increasing worldwide, with implications for serious pregnancy complications.

**Methods:**

Following PRISMA guidelines, a systematic search was conducted in both Chinese and English databases up to December 30, 2020. Pregnancy complications and outcomes including gestational diabetes mellitus (GDM), gestational hypertension (GHTN), pre-eclampsia, cesarean section (CS), induction of labor (IOL), and postpartum hemorrhage (PPH) were analyzed. Random-effects or fixed-effects models were utilized to calculate the odds ratio (OR) with 95% confidence intervals (CIs).

**Results:**

Women with overweight and obesity issues exhibited significantly higher risks of GDM (OR, 2.92, 95%CI, 2.18-2.40 and 3.46, 95%CI, 3.05-3.94, respectively) and GHTN (OR, 2.08, 95%CI, 1.72-2.53 and 3.36, 95%CI, 2.81-4.00, respectively) compared to women of normal weight. Pre-eclampsia was also significantly higher in women with overweight or obesity, with ORs of 1.70 (95%CI, 1.44-2.01) and 2.82 (95%CI, 2.66-3.00), respectively. Additionally, mothers with overweight or obesity issues had significantly higher risks of CS (OR, 1.44, 95%CI, 1.41-1.47, and 2.23, 95%CI, 2.08-2.40), IOL (OR, 1.33, 95%CI, 1.30-1.35 and 1.96, 95%CI, 1.85-2.07), and PPH (OR, 1.67, 95%CI, 1.42-1.96 and 1.88, 95%CI, 1.55-2.29).

**Conclusion:**

Women with overweight or obesity issues face increased risks of pregnancy complications and adverse outcomes, indicating dose-dependent effects.

## Introduction

The prevalence of obesity is skyrocketing across the globe. According to a study on the Global Burden of Disease, the proportion of adults with a body mass index (BMI) of 25 or greater surged from approximately 29% to 37% in men and from around 30% to 38% in women between 1980 and 2013. Additionally, in 2013, 22.6% of girls in developed countries and 13.4% of girls in developing countries were classified as overweight or obesity (1).

Nowadays, the prevalence of obesity among women of reproductive age is on the rise globally (2). In most developed countries, over half of women of reproductive age are classified as overweight (BMI 25-29.9 kg/m^2^) or obesity (≧30 kg/m^2^) (3). It has been estimated that 23.9% of any pregnancy complication was attributable to maternal overweight/obesity (4). M Maternal obesity is associated with a myriad of adverse perinatal outcomes (5), including large for gestational age, macrosomia, preterm birth, and stillbirth (6), Additionally, it can impact delivery outcomes (7), such as cesarean section (CS), induction of labor (IOL), and shoulder dystocia (8), as well as contribute to various pregnancy complications (9), like miscarriage, gestational diabetes mellitus (GDM) (10), etc.

Numerous original studies have extensively examined the impacts of pre-pregnancy BMI on maternal health outcomes (11, 12), Numerous original studies have extensively examined the impacts of pre-pregnancy BMI on maternal health outcomes (13–16). However, these reviews primarily focused on English and French publications and used a limited number of studies to analyze various complications, including maternal, fetal, and neonatal adverse outcomes. Notably, many studies on Chinese women have been published in reputable domestic journals in Chinese (17–19). These studies and their findings may contribute to differences in the relationship between maternal BMI and pregnancy outcomes compared to previous studies. Given the existing literature landscape and the desire for a more focused analysis, this article exclusively examines the relationship between BMI and maternal outcomes. This approach is taken because our previous research has already summarized the relationship between BMI and neonatal or fetal outcomes (5), concentrating solely on maternal outcomes aims to ensure the analysis and discussion are more comprehensive in this paper. Therefore, we conducted a systematic review and meta-analysis to investigate the relationship between maternal pre-pregnancy BMI and the risk of pregnancy complications and outcomes. The pregnancy complications and outcomes evaluated in this meta-analysis include GDM, GHTN, pre-eclampsia, CS, IOL, and PPH.

## Materials and methods

### Search strategy

The Preferred Reporting Items for Systematic Reviews and Meta-analyses (PRISMA) guidelines were followed for conducting the systematic search. The search encompassed various databases from their earliest available dates up to December 30, 2020. Chinese databases, including China National Knowledge Infrastructure (CNKI), Wiper database (VIP), China Biomedical Literature Database (CBM), and Wanfang database (WF), were searched, along with English databases such as PubMed, Embase, and ISI. The search strategy involved identifying relevant literature using the following terms: (“BMI” or “Body Mass Index” or “obesity” or “overweight” or “underweight” or “Quetelet index”) AND (“pregnancy complications” or “outcomes”). Additionally, efforts were made to include unpublished studies to mitigate publication bias.

### Study eligibility

Studies were considered eligible for inclusion in this meta-analysis if they met the following criteria: (1) The study type was observational, including cross-sectional, case-control, or cohort designs. (2) Participants were women, with a measured BMI in the first trimester of pregnancy or at their first antenatal visit. (3) Complete baseline maternal clinical information and pertinent outcome data were available. (4) Participants were singleton pregnant with no pre-existing medical disorders before conception. (5) Studies provided the number of women in each BMI category and reported the occurrence of related adverse outcomes. (6) Exposure groups consisted of women classified as underweight, overweight, or obesity, while the control group comprised women of normal weight. (7) The outcomes of interest were adverse pregnancy complications, which encompassed gestational diabetes mellitus [GDM, defined based on a 75g 2-hour oral glucose tolerance test conducted in the second trimester of pregnancy], gestational hypertension [GHTN, also known as pregnancy-induced hypertension, PIH, defined as diastolic blood pressure ≥90 mm Hg or systolic blood pressure ≥140 mm Hg in the second or third trimester among mothers who had normal blood pressure before pregnancy], and pre-eclampsia [defined as blood pressure ≥140/90 mmHg accompanied by proteinuria], as well as delivery complications, including cesarean section [CS, encompassing both elective and emergency deliveries], induction of labor[IOL, involving the administration of inducing drugs such as prostaglandins or oxytocin to expedite delivery], and post-partum hemorrhage [PPH, defined as blood loss exceeding 500mL within 24 hours after delivery].

Studies were excluded if they involved women with incomplete information on height or weight, or if they did not report any outcomes relevant to the scope of this meta-analysis.

### Data extraction and quality assessment

The following information was extracted from each study: author name, country of population, year of publication, study design, the number of women categorized into different BMI levels, pregnancy outcomes, and their occurrence. Data extraction was performed independently by two authors to ensure accuracy, and any inconsistencies were resolved through consensus or consultation with other authors.

The quality assessment of case-control or cohort studies was conducted using the 9-star Newcastle-Ottawa Scale, while the quality of cross-sectional studies was evaluated using the 11-point scale from the Agency for Healthcare Research and Quality (AHRQ). Each study is based on predefined standards, with a score of ≥7 out of 9 or 9 out of 11 indicating high quality. Studies scoring 5-6 out of 9 or 7-8 out of 11 were classified as medium quality, while those with scores <5 out of 9 or <7 out of 11 were considered low quality. Only studies rated as medium or high quality were included in the analysis. The quality assessment was conducted independently by two authors, with any disagreements resolved through consensus.

### Statistical analysis

BMI, also known as the Quetelet index, is defined as (weight in kilograms)/(height in meters)^2^ (20). It has become a universally accepted measure of the degree of overweight or obesity. The World Health Organization (WHO) and the National Institute of Health (NIH) have defined three cutoff points (18.5 kg/m^2^, 25.0 kg/m^2^, 30.0 kg/m^2^), which classify individuals into four groups: underweight, normal weight, overweight and obesity (21).

For this meta-analysis, BMI levels were categorized into these four groups, with normal weight serving as the reference group. The risk of pregnancy complications for individuals in other BMI categories was assessed using odds ratios (OR) with 95% confidence intervals (CIs).

Heterogeneity among studies was assessed using standard chi-square tests and *I^2^* values, with tests carried out in Stata. A random-effects model (REM) was employed in the presence of heterogeneity (*I^2^* >50%), while the fixed-effect model (FEM) was utilized otherwise. Sensitivity analysis was conducted to examine potential sources of heterogeneity. Additionally, meta-regression was conducted to identify the sources of the heterogeneity. The funnel plot and Egger’s regression asymmetry test were used to assess the potential publication bias.

Data analysis was conducted using Stata version 11.0 (Stata Corporation, College Station, TX, USA) software. All *p*-values were two-tailed, and *p*-value < 0.05 was considered statistically significant.

## Results

### Search results

**Figure 1:**
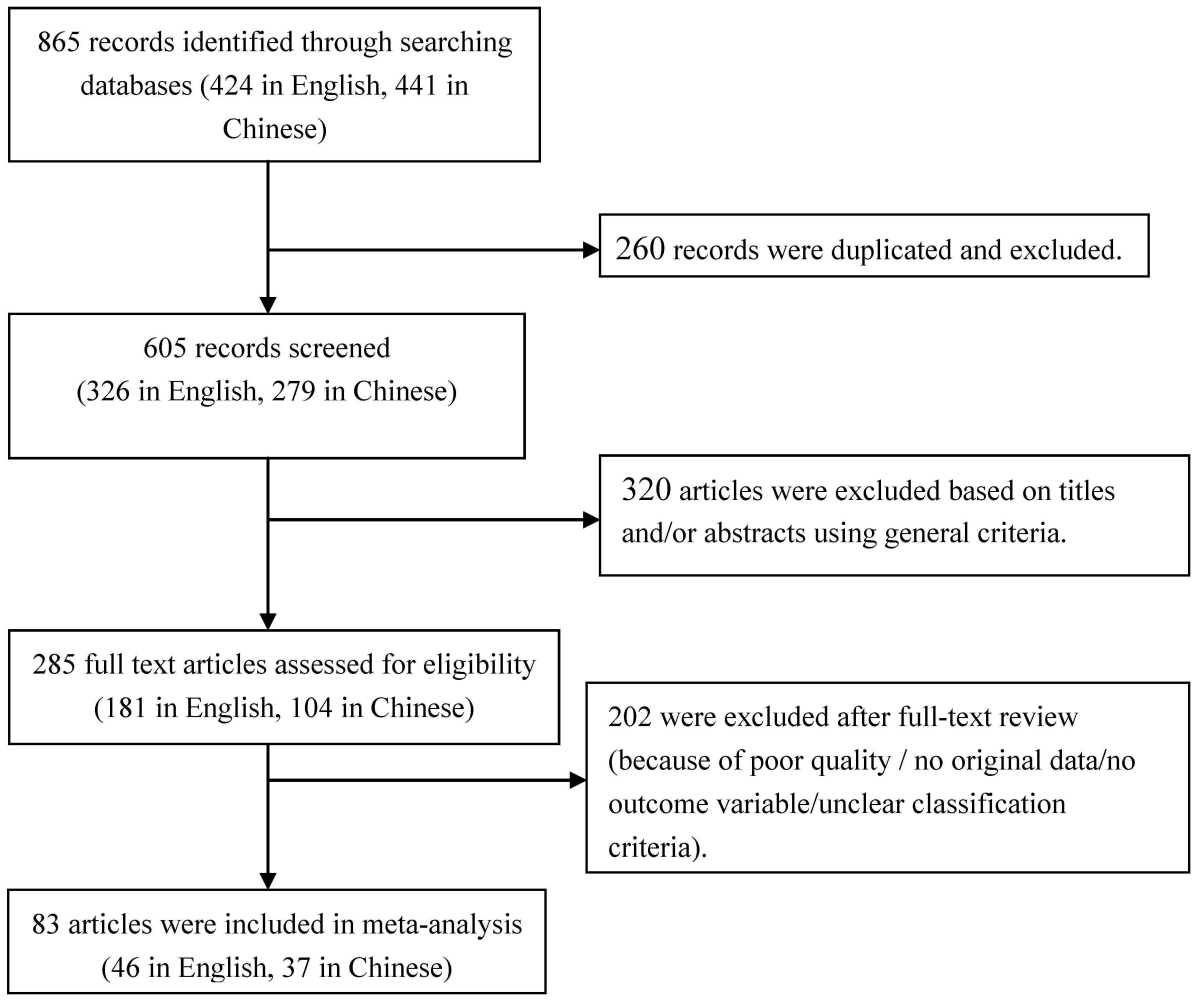
Flow diagram of selecting articles for inclusion.

Initially, 865 articles were identified regarding the association between maternal BMI and pregnancy complications. Of these, 441 were found in Chinese databases and 424 in English databases. After excluding 260 duplicated articles, 605 records remained. Subsequently, 285 articles were selected for full-text review after removing 320 pieces based on screening of titles and abstracts. Eventually, 83 studies met all general criteria and were included in the quantitative analysis. A flow diagram illustrating the selection process is provided in **Figure 1**.

### Characteristics of included studies

These 83 studies, encompassing 1,966,026 women, were published between 1998 and 2019, with sample sizes ranging from 100 to 621,221. The majority of studies were carried out in Asia (n = 53) [China (n = 42), India (n = 5), Korea (n = 2), United Arab Emirates (n = 2), Iran (n = 1), Israel (n = 1)]. Additionally, 16 studies were conducted in Europe [UK (n=6), Denmark (n = 3), Turkey (n=2), Finland (n = 1), Ireland (n = 1), Italy (n = 1), Spain (n = 1), Sweden (n = 1)]. Eight studies were conducted in North America [USA (n=8)], three in Africa [Cameroon (n=1), Nigeria (n=1), Sudan (n=1)], and three in Oceania [Australia (n=3)]. The primary information regarding these studies is summarized in **Table 1**.

**Table 1:** Information of articles included in analysis of association between maternal BMI and pregnancy complications.

Study	Country	Study Design^*^	Underweight	Normal	Overweight	Obese	Quality Score
Ogunyemi D 1998 (20)	USA	C	78	223	78	203	8
Kumari AS 2000 (21)	United Arab Emirates	RC	–	300	–	188	8
Michlin R 2000 (22)	Israel	CC	–	167	–	167	7
Sebire NJ 2001 (23)	UK	RC	–	176923	79014	31276	8
Kaufman H 2001 (24)	USA	RC	6135	11886	4189	2221	9
Baeten JM 2001 (25)	USA	RC	18988	50425	17571	9817	9
Yajun Yuan 2003 (26)	China	RC	545	549	–	544	6
Cedergren MI 2004 (27)	Sweden	PC	–	535900	–	85321	9
Li Ping 2004 (28)	China	PC	158	152	23	–	7
Liu Xuejun 2004 (29)	China	C	28	159	76	17	7
Kristensen J 2005 (30)	Denmark	PC	1812	19169	2573	951	8
Caihong Luo 2005 (31)	China	RC	271	541	–	308	6
Raatikainen K 2006 (32)	Finland	PC	–	20333	3388	1880	8
Roman H 2007 (33)	USA	PC	–	2081	–	2081	9
Smith GC 2007 (34)	UK	C	17968	95516	50214	23592	9
Fu B 2007 (35)	China	RC	63	256	97	34	6
Han Aihong 2007 (36)	China	PC	61	654	262	105	7
Bhattacharya S 2007 (37)	UK	RC	2842	14076	5308	2015	8
Driul L 2008 (38)	Italy	RC	230	533	102	51	8
Zheng Min 2008 (39)	China	RC	97	482	181	79	8
Leung TY 2008 (40)	China	RC	2629	22041	3956	677	7
Joy S 2009 (41)	USA	RC	–	9171	–	3744	9
Khashan AS 2009 (42)	Ireland	C	2581	45463	25476	16203	9
Schrauwers C 2009 (43)	Australia	RC	–	100	100	170	9
Hoff GL 2009 (44)	USA	RC	–	125	568	342	6
Knight M 2010 (45)	UK	C	–	634	–	659	9
Mantakas A 2010 (46)	UK	RC	737	3102	1727	1048	8
Aydin C 2010 (47)	Turkey	RC	–	5685	2214	1213	8
Athukorala C 2010 (48)	Australia	RC	–	943	446	272	7
Xuemei Li 2010 (49)	China	PC	258	1245	394	–	6
Park JH 2011 (50)	Korea	RC	385	1387	539	–	9
Juanhua Tang 2011 (51)	China	PC	52	682	–	66	7
Qiaoying Liu 2011 (52)	China	RC	–	340	–	40	6
Bailing Jiang 2011 (53)	China	RC	67	258	85	–	8
Xuemin Liu 2011 (54)	China	RC	579	3200	926	342	7
Joshi S 2011 (55)	India	C	–	838	251	111	8
Rayis DA 2011 (56)	Sudan	CS	–	654	597	323	7
Han YS 2011 (57)	Korea	C	111	363	67	67	9
Green C 2011 (58)	Australia	RC	–	179	–	45	9
Ezeanochie MC 2011 (59)	Nigeria	CC	–	201	–	201	8
Mandal D 2011 (60)	India	PC	–	422	–	422	8
Ovesen P 2011 (61)	Denmark	RC	15776	233160	77250	43161	9
Situ Wenbei 2011 (62)	China	C	–	140	100	40	8
Meenakshi 2012 (63)	India	PC	–	45	87	83	8
Verma A 2012 (64)	India	PC	116	406	165	97	9
Halloran DR 2012 (65)	USA	RC	–	11308	2388	1425	7
Sebastian 2012 (66)	Spain	RC	168	2597	–	251	8
Jing Xu 2012 (67)	China	RC	247	1452	202	63	7
Jie Chen 2012 (68)	China	RC	64	352	121	78	6
Guohua Meng 2012 (69)	China	RC	157	541	–	60	6
Zhenyu Cai 2012 (70)	China	PC	309	1319	259	70	7
Yazdani S 2012 (71)	Iran	RC	128	412	356	104	7
Jain D 2012 (72)	India	CS	10	188	102	–	7
Xiaofeng Xu 2012 (73)	China	C	66	364	116	54	8
Magann EF 2013 (74)	USA	PC	276	1965	1072	1177	9
Oteng-Ntim E 2013 (75)	UK	C	967	10101	4349	2227	8
Yundi Liu 2013 (76)	China	RC	31	189	–	76	6
Vaswani PR 2013 (77)	United Arab Emirates	RC	–	420	635	930	8
Qingping Zhao 2013 (78)	China	RC	–	50	–	50	6
Jin Tong 2013 (79)	China	RC	445	1685	–	279	7
Nan Li 2013 (80)	China	PC	3809	21942	6185	2037	9
Kai Shi 2013 (81)	China	RC	–	35	31	60	6
Lulu Chen 2014 (82)	China	PC	56	170	–	24	6
Jian Jin 2014 (17)	China	RC	56	157	–	43	6
Gesche J 2015 (83)	Denmark	RC	–	455	–	231	9
Ding XX 2015 (84)	China	PC	2365	7240	–	646	9
Fouelifack FY 2015 (12)	Cameroon	RC	17	228	152	65	8
Lu Liu 2015 (18)	China	RC	250	1370	388	116	8
Rezi Wanguli 2016 (85)	China	RC	132	606	–	112	7
Haiyan Liu 2016 (86)	China	RC	188	509	–	58	7
Aiying Song 2017 (87)	China	RC	150	150	150	150	7
Jianling Tang 2017 (88)	China	RC	40	290	–	50	6
Aozheng Chen 2017 (89)	China	RC	42	795	135	28	7
Jingyuan Lv 2017 (90)	China	RC	108	521	113	38	8
Kansu-Celik H 2017 (11)	Turkey	PC	–	261	–	80	8
Xiuhui Qu 2018 (91)	China	RC	49	526	211	84	7
Yinchun Liu 2018 (92)	China	RC	49	391	–	75	6
Chenxiang Du 2018 (93)	China	RC	75	254	69	30	7
Yuqiao Yang 2019 (94)	China	RC	210	628	–	362	7
Huying Zhao 2019 (95)	China	RC	174	–	196	79	6
Lili Wang 2019 (96)	China	RC	395	901	–	773	7
Jinghua Li 2019 (19)	China	PC	1119	1128	120	27	8
Zhao RF 2019 (97)	China	RC	1687	8123	1149	177	9

*PC, Prospective cohort; RC, Retrospective cohort; C, Cohort; CC, Case-Control; CS, Cross-sectional.

Among the 83 studies, 63 involved CS, 58 involved GDM, 34 involved GHTN, 38 involved PPH, 27 involved pre-eclampsia, and 19 involved IOL. These studies were utilized to assess the risk of pregnancy complications at different BMI levels. The number of studies and individuals involved is presented in **Table 2**.

**Table 2:** The number of articles and individuals of each complication involved in this meta-analysis.

Complication	No.	Under VS Normal			Over VS Normal			Obesity VS Normal		
		No.	Under +/-	Normal +/-	No.	Over +/-	Normal +/-	No.	Obesity +/-	Normal +/-
GDM	58	36	942/30669	7339/298064	29	3644/98773	4873/231082	37	2193/13475	4360/59583
GHTN	34	19	542/8973	3644/36594	15	2204/9342	4572/35850	23	1640/6836	4982/47253
Pre-eclampsia	27	15	744/41155	10235/363066	15	929/22923	1658/62375	15	2025/29799	3477/152086
CS	63	40	9899/37362	44509/117059	31	17029/51141	33096/138224	50	15659/30012	52265/147678
IOL	19	6	961/3437	5710/17894	12	18788/72728	35420/175808	19	22997/104899	78225/683427
PPH	38	24	680/8417	5370/40500	21	1661/11550	4271/38161	30	9669/75427	39104/425626

No, number of studies; GD, gestational diabetes mellitus; GHTN, gestational hypertension; PPH, post-partum haemorrhage; CS, cesarean section including emergent CS and selective CS; IOL, Induction of labor.

### Methodological quality

Eighty-one cohort or case-control studies were assessed using the NOS scale and obtained an average score of 7.58 ± 1.05 (**Table 1**). Among these, 65 were classified as high-quality studies, while 16 were categorized as medium-quality studies. Additionally, two cross-sectional studies were assessed using the AHRQ scale and were deemed of medium quality, each receiving 7 points.

### Maternal pre-pregnancy BMI and the risk of pregnancy complications

**Figure 2:**
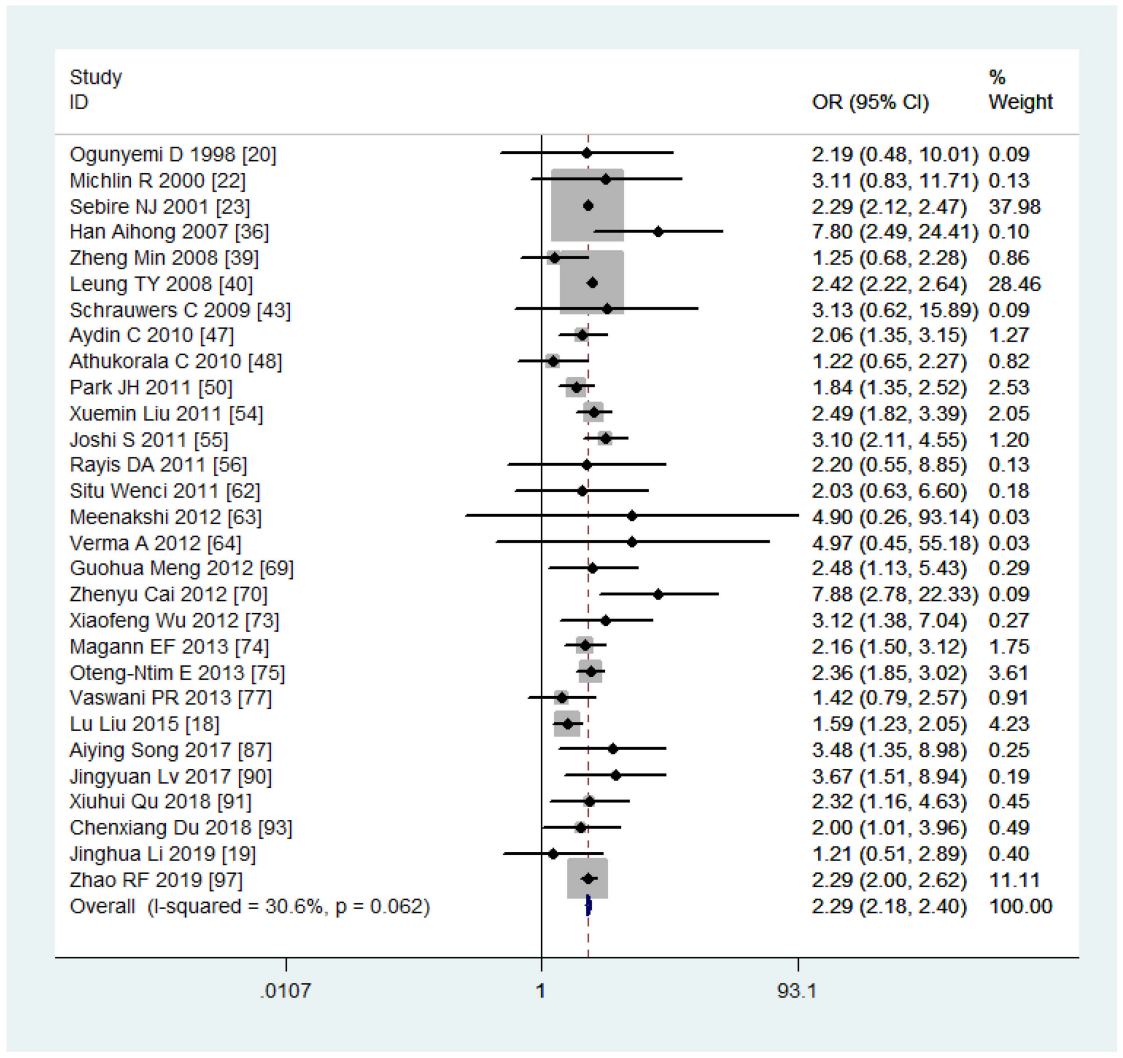
Gestational diabetes mellitus forest plot for overweight compared with normal weight.

**Figure 3:**
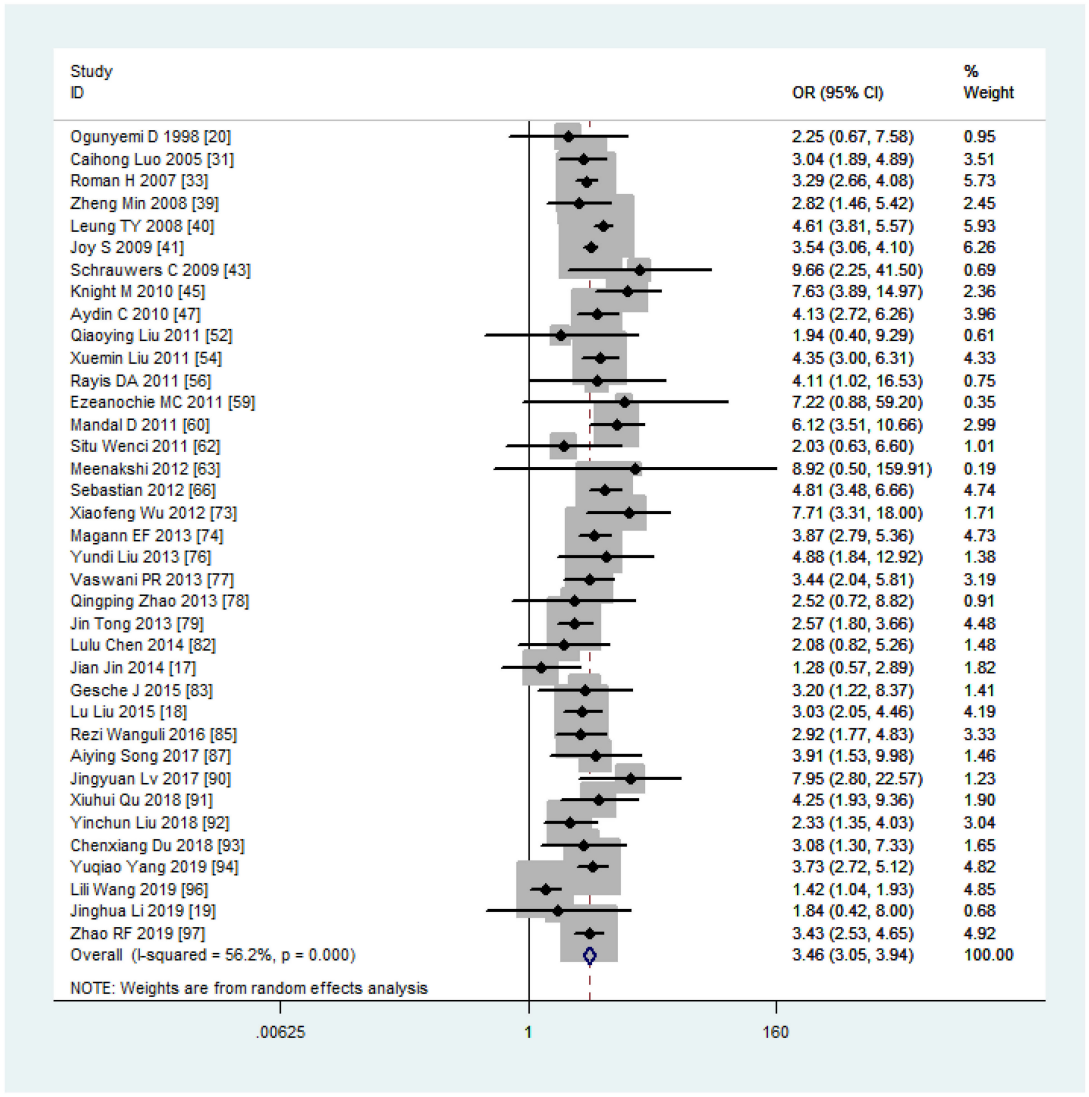
Gestational diabetes mellitus forest plot for obesity compared with normal weight.

**Figure 4:**
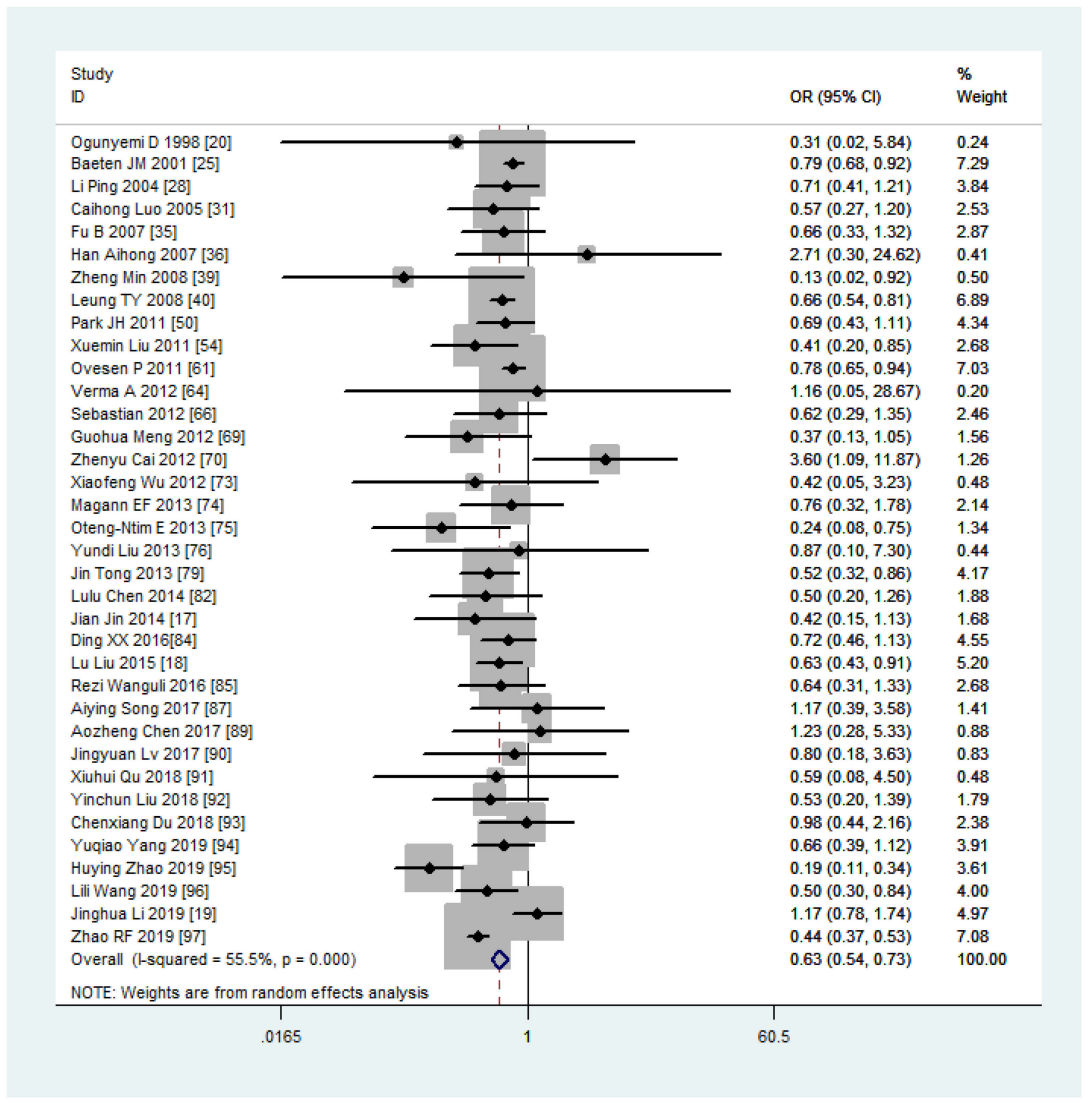
Gestational diabetes mellitus forest plot for under weight compared with normal weight.

Women with overweight and obesity exhibited a significantly higher risk of GDM (OR, 2.92, 95%CI, 218-2.40 and 3.46, 95%CI, 3.05-3.94, respectively, as shown in **Figures 2**, **3**) and GHTN (OR, 2.08, 95%CI, 1.72-2.53 and 3.36, 95%CI, 2.81-4.00, respectively) compared to women of normal weight. Conversely, when women were underweight, these risks were lower (GDM: OR, 0.63, 95%CI, 0.54-0.73, as depicted in **Figure 4**; GHTN: OR, 0.64, 95%CI, 0.58-0.71). Pre-eclampsia, a complication of GHTN, was significantly more prevalent in women with overweight and obesity, with ORs of 1.70 (95%CI, 1.44-2.01) and 2.82 (95%CI, 2.66-3.00). On the other hand, pre-eclampsia was significantly lower in women classified as underweight, with an OR of 0.69 (95%CI, 0.64-0.75).

**Figure 5:**
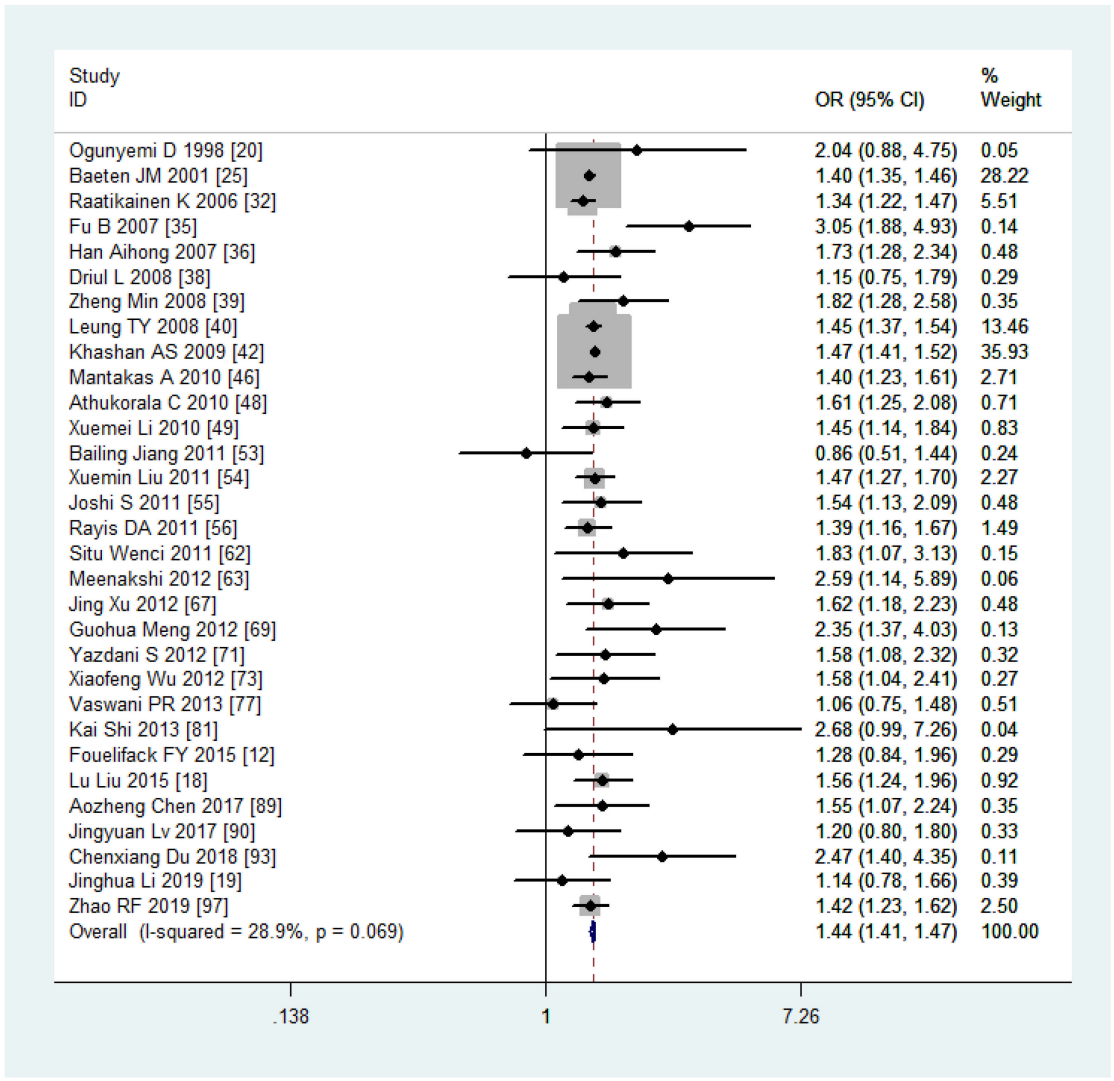
Cesarean section forest plot for overweight compared with normal weight.

Analyzing the mode of delivery in mothers with overweight and obesity revealed a significantly higher risk of CS (OR, 1.44, 95%CI, 1.41-1.47, as depicted in **Figure 5**, and 2.23, 95%CI, 2.08-2.40) and IOL (OR, 1.33, 95%CI, 1.30-1.35 and 1.96, 95%CI, 1.85-2.07), respectively. In contrast, mothers classified as underweight had a significantly lower risk of CS (OR, 0.75, 95%CI, 0.73-0.77). Women with overweight and obesity had higher odds of PPH (OR, 1.67, 95%CI, 1.42-1.96 and 1.88, 95%CI, 1.55-2.29) while being underweight was associated with lower odds (0.67, 95%CI, 0.62-0.73). The ORs, heterogeneity, and selected pooling models are presented in **Table 3**.

**Table 3:** The associations between maternal pre-pregnancy BMI and pregnancy complications and outcomes.

Outcome	Under VS Normal			Over VS Normal			Obesity VS Normal		
	*I^2^* (%)	Models	OR (95%CI)	*I^2^* (%)	Models	OR (95%CI)	*I^2^* (%)	Models	OR (95%CI)
GDM	55.5	REM	**0.63(0.54-0.73)**	30.6	FEM	**2.92(2.18-2.40)**	56.2	REM	**3.46(3.05-3.94)**
GHTN	39.9	FEM	**0.64(0.58-0.71)**	77.7	REM	**2.08(1.71-2.53)**	71.0	REM	**3.36(2.81-4.00)**
Pre-eclampsia	30.8	FEM	**0.69(0.64-0.75)**	56.6	REM	**1.70(1.44-2.01)**	39.4	FEM	**2.82(2.66-3.00)**
CS	29.4	FEM	**0.75(0.73-0.77)**	28.9	FEM	**1.44(1.41-1.47)**	72.5	REM	**2.23(2.08-2.40)**
IOL	54.3	REM	0.94(0.79-1.11)	1.9	FEM	**1.33(1.30-1.35)**	66.0	REM	**1.96(1.85-2.07)**
PPH	31.5	FEM	**0.67(0.62-0.73)**	56.9	REM	**1.67(1.42-1.96)**	95.2	REM	**1.88(1.55-2.29)**

The bold values represent the confidence intervals, which exclude 1, indicating statistical significance.

### Sensitivity analysis

**Figure 6:**
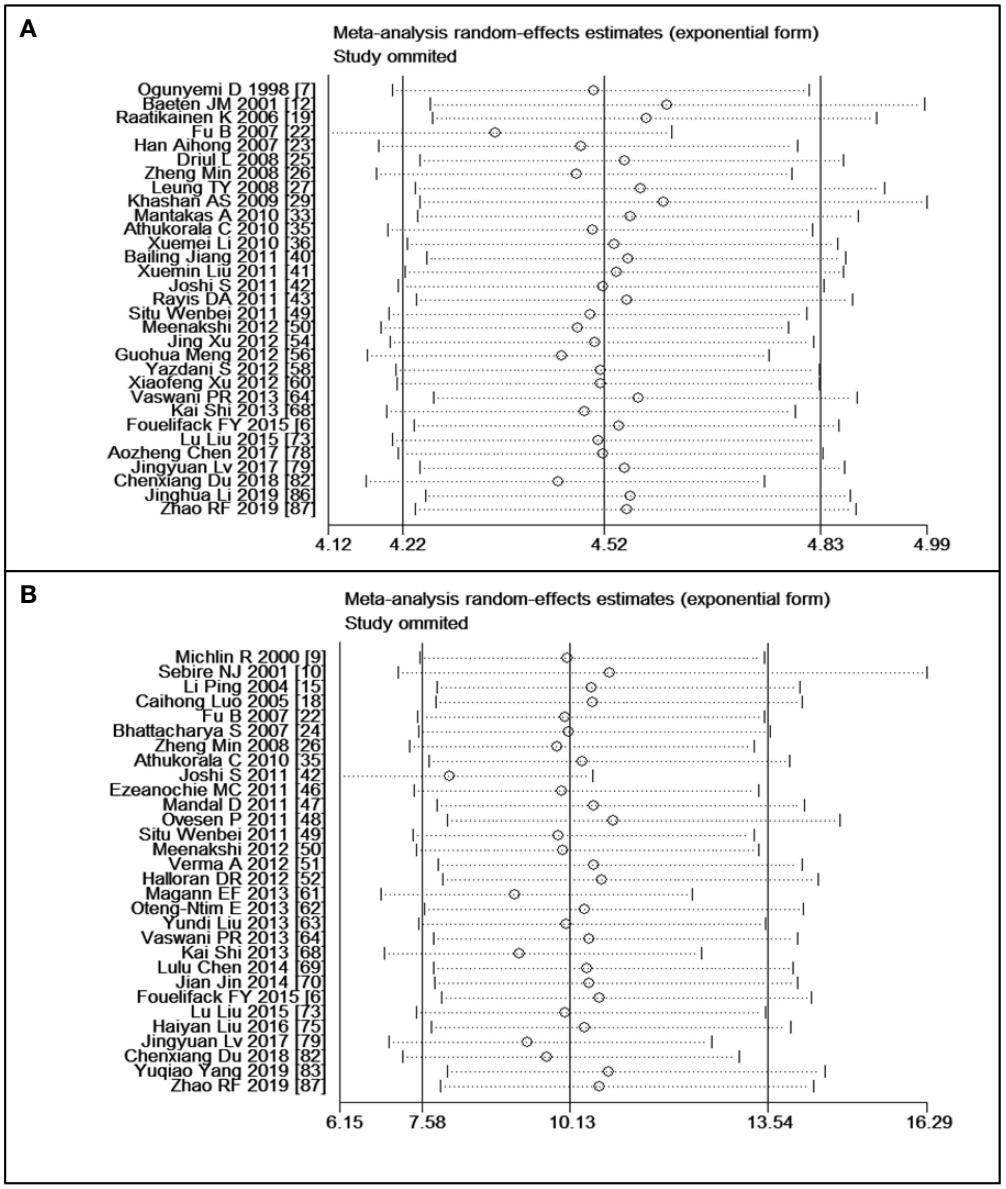
Sensitivity analyses: **(A)** overweight versus normal weight of cesarean section, **(B)** obesity versus normal weight of postpartum haemorrhage.

Sensitivity analyses were performed following the summary effect of each of the six outcomes, particularly when significant heterogeneities (*I^2^* > 50%) were observed. In such cases, influential studies were identified and excluded to reassess the combined effect. For example, in the analysis comparing women who were overweight versus those with normal weight in the CS variable, the original *I^2^* value was greater than 76.4%. After removing three articles, the value dropped to 28.9%. The results of sensitivity analyses are shown in **Figure 6A**. As indicated in **Table 3**, the majority of results exhibited low or moderate heterogeneity, except for the comparison of obesity versus normal in PPH. For this outcome, sensitivity analyses were illustrated in **Figure 6B**.

### Sub-group analysis and meta-regression

Due to the high heterogeneity observed in PPH among mothers with obesity, we conducted subgroup and meta-regression analyses to explore the source of this heterogeneity. Among the 30 studies included in the analysis, 16 were conducted in China, and 22 were conducted in Asia. Therefore, we performed subgroup analyses and meta-regression using the country and continent of study to assess their potential contributions to the observed heterogeneity in the studies.

**Figure 7:**
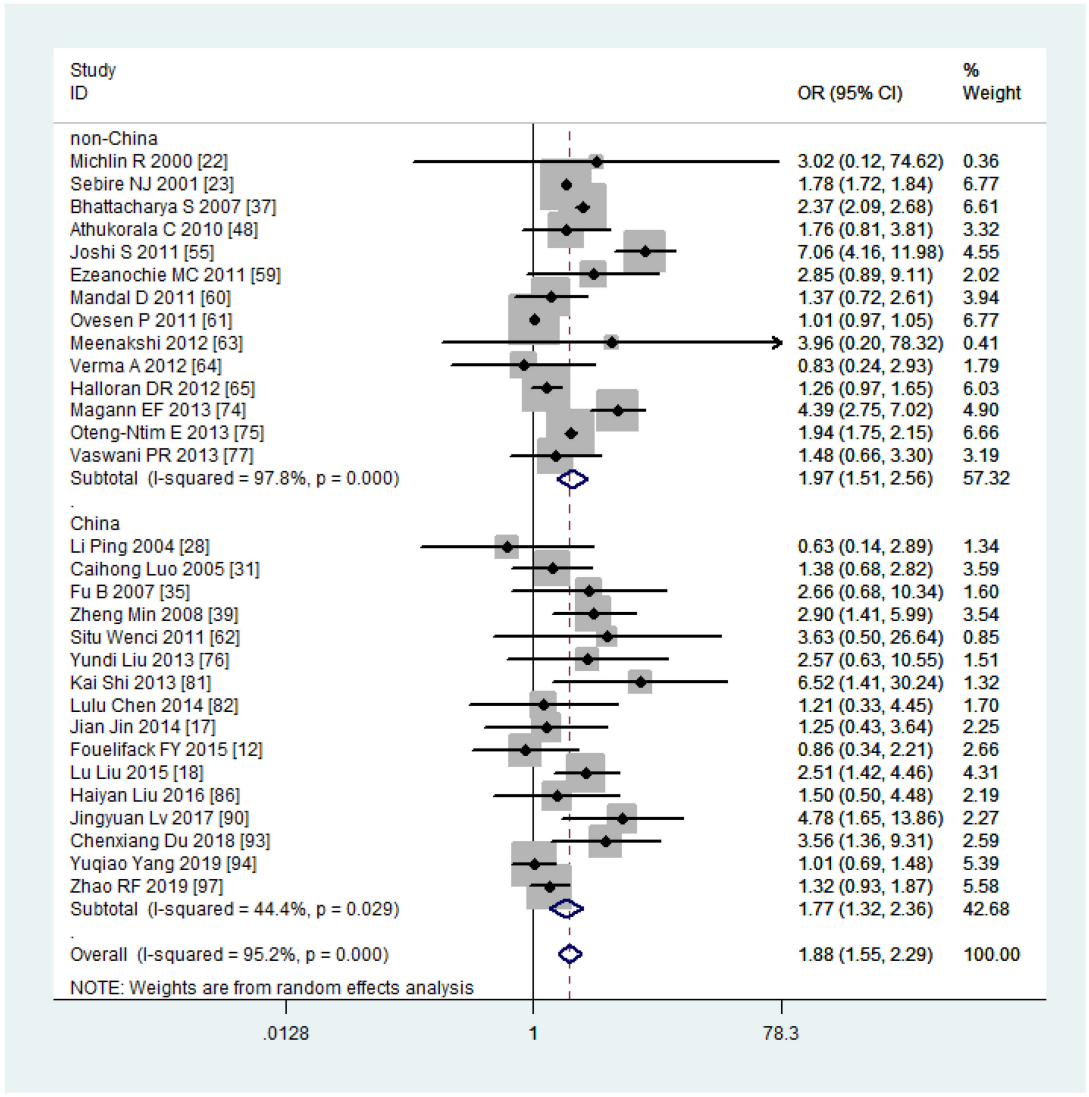
Sub-group analysis (China VS non-China) of Postpartum haemorrhage for overweight compared with normal weight.

**Figure 8:**
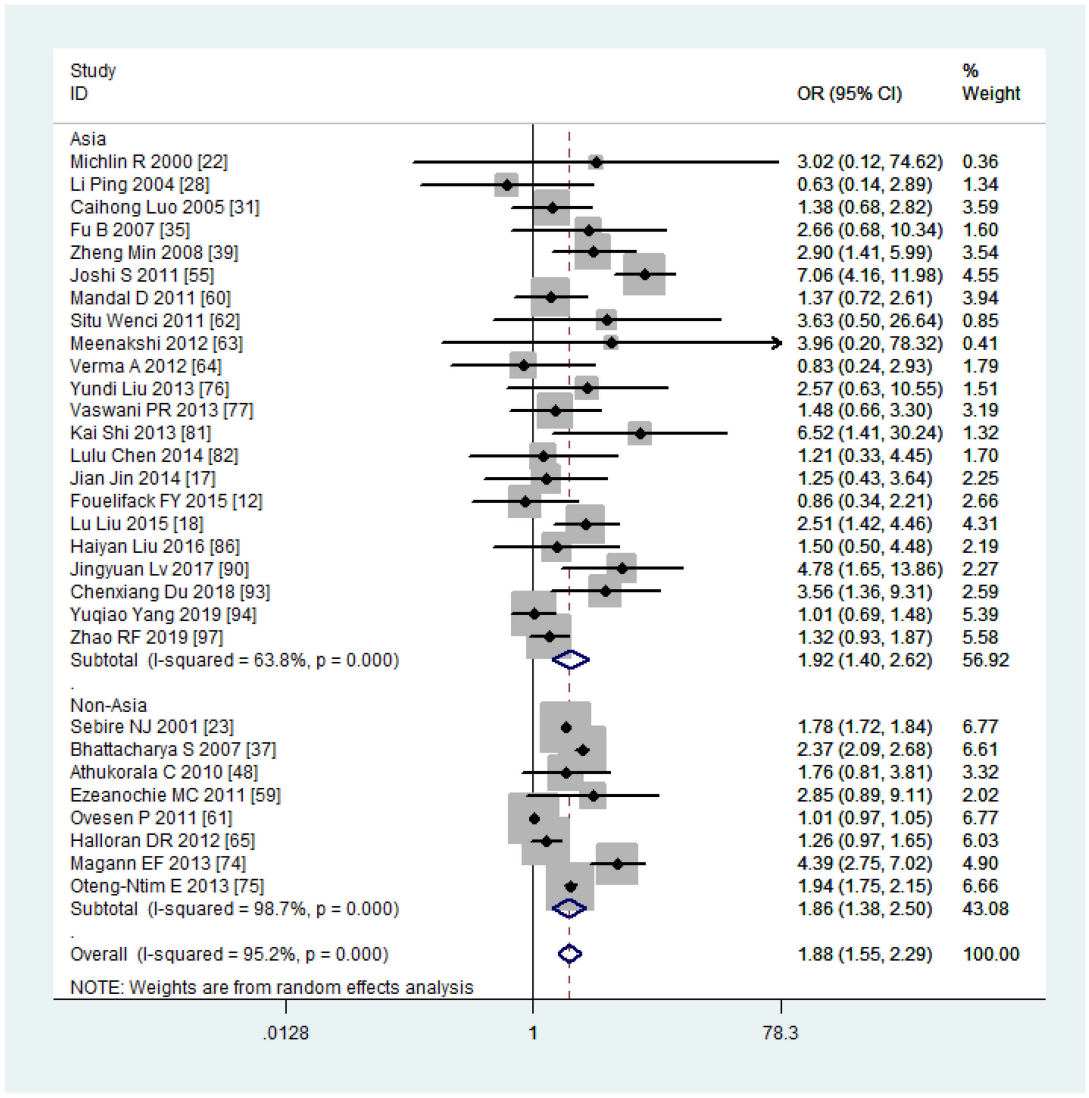
Sub-group analysis (Asia VS non-Asia) of Postpartum haemorrhage for overweight compared with normal weight.

The subgroup analysis revealed that the risk of PPH was lower among mothers with obesity in China (OR, 1.77, 95% CI, 1.32-2.36, with *I^2^ = *44.4%) compared to other countries (OR, 1.97, 95% CI, 1.51-2.56, with *I^2^ = *97.8%) (**Figure 7**). When stratified by continent of study, mothers with obesity had a higher risk of PPH in Asia (OR, 1.92, 95% CI, 1.40-2.62, with *I^2^ = *63.8%) compared to non-Asia regions (OR, 1.86, 95% CI, 1.38-2.50, with *I^2^ = *98.7%) (**Figure 8**). Although the heterogeneity was lower in the China or Asia subgroup analyses by countries or continent, respectively, the OR value of the Asian group was greater than that of China. This difference may be attributed to one study conducted in India among the six articles in Asia but not in China, with a reported OR value of 7.06 (55), thereby elevating the overall OR value in Asia. Meta-regression analysis indicated that neither the country nor the continent of study was the source of heterogeneity, with *p-*values of 0.502 and 0.416, respectively. It is plausible that other factors such as the age and parity of pregnant women, sample size, season of pregnancy, or environmental factors may contribute to the observed significant heterogeneity.

Despite all studies being observational, among them there are two case-control studies and two cross-sectional studies, we attempted to perform subgroup analysis based on the study design. Results indicate that for the CS outcome, the OR values of case-control or cross-sectional studies have slightly decreased compared to the previous total results, yet the 95% confidence interval has significantly widened. Conversely, for cohort studies, there is minimal difference between the results and the total results. Due to the limited number of articles by case-control and cross-sectional study designs for other outcomes, subgroup analysis was not conducted.

### Publication bias evaluation

**Figure 9:**
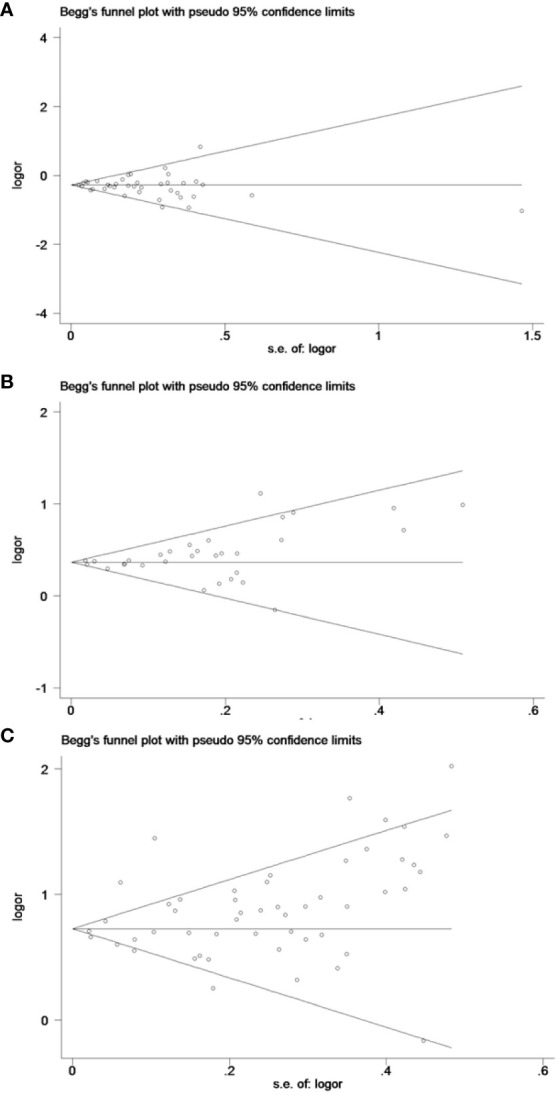
The funnel plots for cesarean section: **(A)** underweight versus normal weight, **(B)** overweight versus normal weight, and **(C)** obese versus normal weight.

The funnel plots and Egger’s test results indicated no significant publication bias (*P* > 0.05) across the 18 results of the six pregnancy outcomes. A funnel plot for CS, which encompassed the largest number of studies, is provided in **Figure 9**.

## Discussion

### Main findings

This study provided a quantitative estimation of the risk of adverse pregnancy complications among mothers with varying BMI levels. It was found that mothers who were diagnosed as overweight or obesity faced a significantly higher risk of pregnancy complications, including GDM, GHTN, and pre-eclampsia. Additionally, they were at a heightened risk of adverse pregnancy outcomes such as CS, IOL, and PPH. Moreover, a dose-dependent relationship was observed, indicating an increased risk as the BMI levels rose.

### Strengths and limitations

While previous systematic reviews and meta-analyses have explored the association between maternal body mass index and maternal health outcomes (13–16), each has its unique approach and findings. Three reviews among them only included 49 (13), 22 (14) and 13 (15) studies, respectively, a limited number in the quantitative synthesis. Furthermore, they all focused on the impact of maternal pre-pregnancy body mass index on maternal, fetal, and neonatal adverse outcomes, which results in less relevant literature on maternal outcomes. For example, the meta-analysis published in 2008 (13), which included 49 articles, only focused on hemorrhage and infection outcomes of pregnant women, with 3-4 studies. In another meta-analysis published in 2019 (15), only five and seven articles were used to analyze the relationship between gestational diabetes, gestational hypertension, and maternal pre-pregnancy BMI. The most extensive review to date, published in 2021 (16), included 86 studies and evaluated the relationship between maternal, fetal, and neonatal adverse outcomes and maternal pre-pregnancy body mass index, a broader range of outcomes. However, while examining almost the same number of studies, our review offers a distinct perspective by including a more diverse range of studies. Particularly, we included more articles from Asia, Africa, Europe, and North America providing a more comprehensive understanding of the global landscape of maternal health outcomes related to BMI. Thus, our findings complement existing literature and offer valuable insights into the worldwide situation.

Our study employed rigorous methodology, conducting comprehensive literature searches and applying stringent inclusion criteria, resulting in the inclusion of 83 articles for quantitative synthesis. The quality of included studies was assessed using the NOS tool for 81 cohort or case-control studies and ARHQ for cross-sectional studies, ensuring methodological robustness. Notably, our analysis revealed medium to low levels of heterogeneity between studies, and the relatively narrow confidence intervals further strengthened the reliability of our findings. These methodological strengths enabled us to draw firm conclusions from our meta-analysis.

There are several limitations to acknowledge in this meta-analysis. Firstly, the majority of included studies relied on pre-pregnancy BMI, with only a small portion using first-trimester BMI. While this discrepancy could potentially impact our meta-analysis results, previous stratified analyses have suggested that the difference may not be statistically significant (14). Secondly, our analysis focused solely on the association between maternal pre-pregnancy BMI and pregnancy outcomes, overlooking the effect of gestational weight gain (GWG). Given that approximately half of reproductive-age women have overweight or obesity issues and are at higher risk of substantial weight gain during pregnancy, the omission of GWG could be a limitation. Indeed, studies have shown that excessive GWG is associated with an increased risk of GHTN, PPH, and CS compared to women with normal weight gain (98). Several recently published meta-analyses (99) have focused on the association between GWG and maternal and infant outcomes. Combining these findings with our results could provide a more comprehensive understanding of the factors influencing pregnancy outcomes. Thirdly, the lack of detailed parity or age data across BMI groups in most studies may introduce bias into the pooled risk estimates. However, many included studies accounted for parity or age imbalances among BMI groups through adjustments during data analysis, mitigating potential biases to some extent. Fourthly, the outcome of CS encompassed both emergent and selective CS, with unclear distinctions provided in many included articles. Considering previous studies indicating that pregnant women with obesity or overweight have a higher likelihood of choosing selective CS over emergent CS (15), this ambiguity could affect our findings. Lastly, despite we have conducted subgroup analyses on certain factors regarding the outcomes of PPH or CS, the absence of subgroup analyses based on regions, study design types, or relevant environmental factors related to other outcomes of interest may constrain the generalizability of our results.

### Interpretation

In our paper, pregnant women with overweight and obesity faced an increased risk of developing GDM, with ORs 2.20 (95% CI, 2.02-2.39) and 3.46 (95% CI, 3.05-3.94), respectively. These findings presented narrower confidence intervals compared to a previous meta-analysis based on the PubMed database and 20 articles conducted in 2007 (10), where the ORs were reported as 2.14 (95% CI, 1.82-2.53) and 3.56 (95% CI, 3.05-4.21). Markedly, our meta-analysis, including 58 studies on GDM, demonstrated low to medium between-study heterogeneities (*I* ^2 = ^54.3%, 33.4%, 56.2%), contributing to the narrowed 95% CI of the ORs. Additionally, research has indicated (100) that the cumulative incidence of type 2 diabetes increased significantly in the first 5 years postpartum due to elevated fasting glucose levels during pregnancy. Therefore, targeting pregnant mothers with elevated glucose levels may represent a more effective approach to diabetes prevention.

In our analysis of 34 studies, it was evident that mothers with overweight and obesity faced an elevated risk of GHTN. While an earlier study suggested that obesity might not independently contribute to pregnancy-induced hypertensive disorders (101), our findings underscored the disparities in GHTN risks across various BMI levels, potentially attributed to higher booking blood pressure among women with obesity (102). Controlling appropriate pre-pregnancy weight could be a preventive measure for GHTN, highlighting the importance of further research to elucidate its underlying mechanisms.

An evident dose-dependent effect was observed concerning maternal pre-pregnancy BMI and the likelihood of delivery by CS. Women with overweight and obesity have a higher propensity to opt for CS (containing emergent CS and selective CS) or IOL, a finding consistent with several previous meta-analyses (16, 103). The heightened risk of CS in women with higher BMI may be attributed to various factors. Firstly, increased BMI could contribute to labor induction failure (104), a potentially leading to CS instead of IOL (103). Moreover, factors such as fetal macrosomia, labor dystocia due to increased pelvic soft tissue, and other complications might further predispose women with higher BMI to CS (61). Furthermore, considering other adverse pregnancy complications like gestational hypertension, gestational diabetes, and fetal complications such as stillbirth or admission to the neonatal intensive care unit (6), these factors could contribute to the increased CS rate observed in women with obesity.

In contrast to women with overweight and obesity, those classified as underweight exhibited a protective effect against GDM, GHTN, pre-eclampsia, CS, and PPH. However, it’s important to note that being underweight during pregnancy may carry its own set of risks, such as an increased likelihood of preterm birth and delivering an SGA or LBW baby (5). Therefore, it is imperative to establish appropriate clinical guidelines and implement public health interventions aimed at managing the weight of pregnant women, whether they are classified as obesity or underweight, in order to safeguard the health of both mothers and their babies.

## Conclusion

Our analysis provides a quantitative estimation of the detrimental effects of pre-pregnancy overweight and obesity on maternal complications and delivery outcomes. Future studies should strive to explore more effective strategies to mitigate the growing threat of overweight and obesity among pregnant women.

## Data availability statement

The original contributions presented in the study are included in the article/**Supplementary Material**. Further inquiries can be directed to the corresponding authors.

## Author contributions

YZ: Writing – original draft, Funding acquisition, Conceptualization, Resources. ML: Writing – original draft, Data curation, Software, Visualization. YY: Writing – original draft, Software, Formal analysis. LX: Writing – review & editing. RZ: Writing – review & editing. CL: Writing – review & editing, Supervision. PL: Writing – original draft, Conceptualization.
